# Evaluation of the statutory surveillance system for invasive MRSA infections in Germany, 2016–2017

**DOI:** 10.1186/s12889-018-5971-y

**Published:** 2018-08-24

**Authors:** Viktoria Schönfeld, M. Diercke, A. Gilsdorf, T. Eckmanns, J. Walter

**Affiliations:** 1 Department for Infectious Disease Epidemiology, Robert Koch Institute, Berlin, Germany; 2 Postgraduate Training for Applied Epidemiology, Robert Koch Institute, Berlin, Germany; 3 European Programme for Intervention Epidemiology Training, ECDC, Stockholm, Sweden

**Keywords:** Disease notification, Program evaluation, Methicillin-resistant *Staphylococcus aureus*, Telephone interviews

## Abstract

**Background:**

Mandatory notification of invasive methicillin-resistant *Staphylococcus aureus* (MRSA) infections was introduced for laboratories in Germany in 2009. The aims were to support local health authorities (LHAs) in their mandate to prevent and control infections in hospitals and to improve population-based nationwide surveillance of healthcare associated infections. We evaluated the MRSA surveillance system to assess whether its aims were met and to identify areas for improvement.

**Methods:**

Using the updated guidelines for evaluating public health surveillance systems by the Centers for Disease Control and Prevention we assessed the attributes simplicity, timeliness, data quality, acceptability, and usefulness. In 2016/2017 we interviewed staff in LHAs, state health authorities (SHAs), and laboratories and analyzed surveillance data of cases notified between 2009 and 2016.

**Results:**

We interviewed 10% of LHAs (*n* = 38), 63% of SHAs (*n* = 10), 5 selected laboratories and analyzed information on 27,706 notified MRSA cases. LHAs reported that on receiving notifications from laboratories they contacted hospitals for clinical information, which was time-consuming and complicated as physicians were hard to reach or refused to answer questions, citing doctor-patient confidentiality. LHAs suggested reducing the amount of information collected as some clinical information was unnecessary for implementing control measures. LHAs stated that they received notifications on time, however surveillance data analysis showed some delay. Data completeness exceeded 90% for most variables, however it was only 68% and 80% for dates of disease onset and hospital admission respectively making it impossible to discriminate between hospital and community acquired infections in half of the cases. The surveillance system was well accepted by half of the interviewees. A third however stated that the benefits of the surveillance system were outweighed by the work associated with it. The majority rated the system to be useful for recognizing trends in the MRSA incidence and the ability to check up on infection control measures in hospitals.

**Conclusions:**

The surveillance system proved to be useful by fulfilling its aims. It was timely, acceptable and provided complete data for most variables. However, the system was complicated; ensuring that only relevant variables are reported could simplify the system without losing any of its usefulness.

## Background

Methicillin-resistant *Staphylococcus aureus* (MRSA) is the multidrug-resistant pathogen that most frequently causes hospital acquired infections in Germany [1, 2]. One mechanism to control communicable diseases is the implementation of effective surveillance systems. Surveillance enables the identification of temporal and regional trends, the evaluation of the effects of interventions, and thereby allows the implementation of effective infection prevention and control (IPC) measures and raises awareness of public health problems [3].

Germany introduced mandatory notification for invasive MRSA infections for laboratories in 2009. The new MRSA surveillance was integrated into the statutory notifiable diseases surveillance system. The aims of the MRSA surveillance are to improve nationwide surveillance of healthcare associated infections by introducing population-based MRSA surveillance and to strengthen local health authorities (LHAs) in their capacity to effectively prevent and control healthcare associated infections [4]. However, it has been questioned whether or not this surveillance system actually contributes to the prevention of nosocomial infections [5]. In addition, periodical evaluations of surveillance systems are crucial in order to ensure that systems fulfill their aims and to implement improvements as required [6–8]. The German surveillance system for MRSA has not yet been evaluated in detail.

We evaluated the surveillance system for invasive MRSA infections by analyzing surveillance data and by interviewing key users of the system including staff in LHAs, state health authorities (SHAs), and laboratories to assess whether aims of the system are being met and to identify areas for potential improvement.

## Methods

### Evaluation

We used methods described in the updated guidelines for evaluating public health surveillance systems by the Centers for Disease Control and Prevention (CDC), which we modified to fit the surveillance system under evaluation [8] (Table 1). We engaged key stakeholders (at SHAs, LHAs, and the national public health institute, the Robert Koch-Institute (RKI)) in the development of the study design and of a questionnaire. We assessed the attributes simplicity, data quality, timeliness, acceptability, and usefulness. The evaluation was divided into two parts. First, we interviewed staff from LHAs, SHAs, and laboratories using a semi-structured questionnaire, which contained open and closed questions. Second, we analyzed surveillance data of cases notified between 2009 and 2016.

**Table 1:** Methods and measures used for assessing attributes of statutory MRSA surveillance, Germany 2016/2017 (based on CDC guidelines)

Attribute	Methods	Measures and questions used to assess the attribute
Simplicity	Interviews	• Description of the surveillance system • Amount and type of data on cases (for case definition and other data) • Method of data collection including number and types of reporting sources • Time for collecting and managing the data (e.g. data entry)
Timeliness	Data analysis and interviews	• Time elapsed between diagnosis and notification and between notification and data transmission
Data quality	Data analysis and interviews	• Completeness of surveillance data • Presence of data quality checks
Acceptability	Data analysis and interviews	• Quantitative measures: reporting rate, data completeness, completion rate of investigations, timeliness • Perceived public health relevance of disease • Dissemination of aggregated data to interested parties • Assessment of effort in relation to usefulness of the system • Statutory requirements for reporting and data collection
Usefulness	Interviews	• Does the system contribute to a timely implementation of prevention and control measures? • Does the system provide estimates for morbidity? • Does the system detect trends? • Does the system lead to improved clinical practices? • Assessment of usefulness on 1 to 10 scale

### Selection of participants and interviews

We recruited SHAs by asking them at a meeting whether they would participate in the study and sent out information afterwards. In order to recruit LHAs, we asked SHAs to forward an invitation and information sheet about the study to the LHAs in their federal states. We planned to interview 2 to 5 LHAs per federal state depending on the population size. If more than the planned number of LHAs per state were interested in participating in the study, we selected LHAs by including a balanced number of LHAs in urban and rural districts, with small and large population sizes, and with high and low incidence of notified invasive MRSA infections. In addition, LHAs were asked to provide names of notifying laboratories in their district during the interviews and a convenience sample of five laboratories from differently sized districts was scheduled for interviews.

The semi-structured phone interviews with representatives of LHAs, SHAs and laboratories were conducted by two researchers where possible. Interviewees were asked for consent to be re-contacted in case a clarification of their responses was required.

### Data analysis

Data analysis was conducted for all electronically transmitted cases of invasive MRSA infections notified to the Robert Koch Institute between 2009 and 2016 which met the surveillance case definition. Data were extracted from the electronic surveillance database SurvNet@RKI [9] on March 2nd, 2017 and subsequently analyzed using Excel (Microsoft Excel 2010) and Stata (StataSE 14). Data was considered to be complete if “yes”, “no”, or a date was specified for a variable. If LHAs had indicated that a result was either not investigated or could not be ascertained, data was considered to be incomplete.

### Confidentiality and ethical statement

Participation in the interviews was voluntary and interviewees’ personal data (name of interviewee, name of LHA, federal state, etc.) were pseudonymized. Surveillance data contained only variables as required by the German Infection Protection Act. Only aggregated data are published in the results.

## Results

Between June 2016 and January 2017, we interviewed representatives of 38 LHAs (10% of all LHAs), 10 SHAs (63% of all SHAs), and 5 laboratories. Furthermore, we analyzed data of 27,706 cases of invasive MRSA infection notified between 2009 and 2016.

### Description of the system

The surveillance system for invasive MRSA infections is integrated into the statutory notification system regulated by the German Infection Protection Act. It is a comprehensive, passive, case-based surveillance system requiring laboratories to notify MRSA detected in blood or cerebrospinal fluid (CSF). The case definition includes laboratory-confirmed cases irrespective of the clinical presentation [10]. Laboratory confirmation requires the culture of *S. aureus* from blood or CSF and the detection of methicillin resistance either by susceptibility testing or detection of the *mecA* gene [11].

**Fig. 1:**
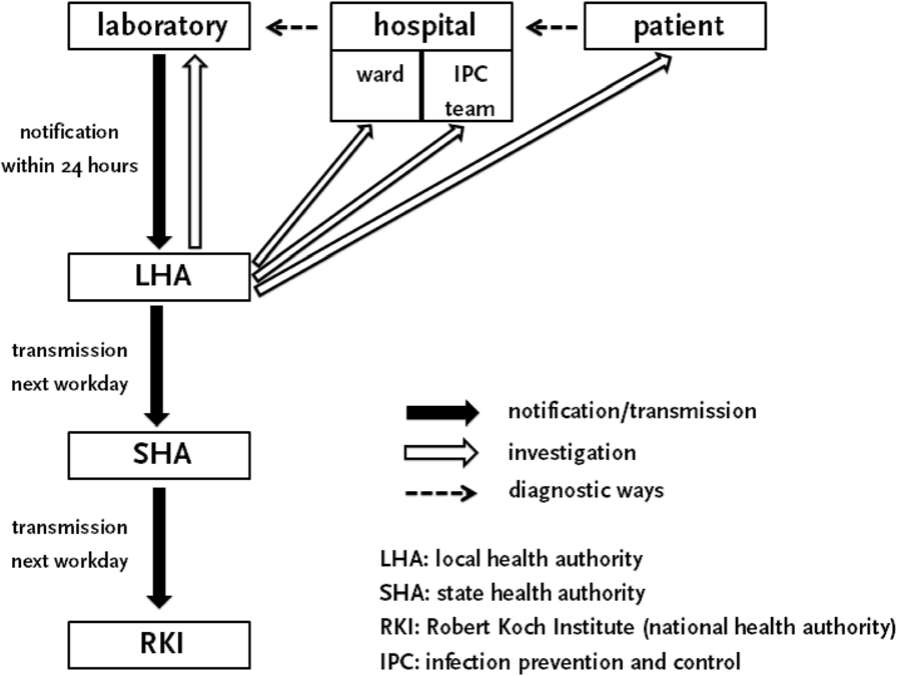
Flow-chart on notification and case investigation processes for laboratory notifications of hospital patients according to the German Infection Protection Act

LHAs should be notified by laboratories about a case of invasive MRSA infection within 24 h. Notifications are usually paper-based and must include but are not limited to demographic information on the patient as well as type and result of the diagnostic tests. LHAs then enter the data into an electronic database and transmit pseudonymized data supplemented by information on clinical presentation and outcome, date of disease onset, and mode of transmission electronically to their SHA within one workday. If available, additional information on the focus of the infection such as invasive devices and catheters, trauma, or other localizations of MRSA infections should also be collected and transmitted. The data is then transmitted electronically by the SHA to the RKI within the next workday (Fig. 1) [12].

According to the Infection Protection Act, laboratories are obliged to make the notification to the LHA responsible for the district from where the sample was sent to the laboratory, which is usually a hospital. The Infection Protection Act also states that the LHA responsible for the district of the patient’s residence is in charge of transmitting the data to their SHA. If the patient’s residence and the hospital are in two different districts, the LHA receiving the notification and the LHA in charge of transmitting the data to the SHA are not the same. In this situation the first LHA (hospital-LHA) has to forward the notification to the second LHA (patient-LHA). There is no legal regulation as to which LHA has to investigate clinical presentation and focus of infection with the hospital.

### Simplicity

All laboratories reported the notification process to be simple and quick as it had been largely automatized. Notification forms were mostly faxed or, by one laboratory, sent by mail to the LHA. Once the notification form had arrived at the LHA, data was entered into the electronic database. Subsequently either health inspectors (73%; 27 of 37 LHAs), physicians (16%; 6 of 37 LHAs), or other staff members (11%; 4 of 37 LHAs) collected information that had not been available in the laboratory notification form. This included variables such as clinical presentation or the focus of the MRSA infection. They also checked whether prevention and control measures had been implemented in the hospital. Specific data collection forms for the investigation of invasive MRSA infections were used by 84% of LHAs (32 of 38). These were either sent to the hospital or used during a telephone interview. LHAs investigated either by email, mail, or fax (51%; 19 of 37) or by phone (49%; 18 of 37). People contacted for this purpose included the treating physician (92%; 34 of 37), the IPC team of the hospital (76%; 28 of 37), and the patients and their families (16%; 6 of 37) (multiple answers were possible). About a third of LHAs (36%; 13 of 36) reported that some hospitals in their districts sent all the required information on their own initiative after they had been alerted by the laboratory about a case of an invasive MRSA infection without requiring any further queries from the LHAs.

**Fig. 2:**
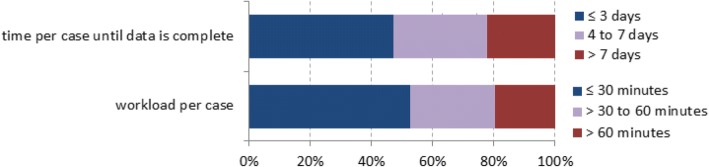
Time spent by LHAs on case investigations of notified MRSA cases; interviews with LHAs 2016/2017, Germany (*n* = 36)

Applying the case definition was perceived as simple by most of the interviewees in LHAs; some reported that there had been some difficulties during the initial phase of the surveillance. Two LHAs stated that health inspectors found it difficult to interpret laboratory reports and that in their opinion physicians should conduct the investigations with laboratories and hospitals. Three LHAs reported that the clinical presentation or the cause of death was not always clear. About half of the LHAs reported investigations to take longer than 30 min and data collection longer than 3 days to complete (Fig. 2). They reported the following reasons for delays in completing the investigations: physicians in the hospitals were hard to reach, especially if hospitals in other districts were involved; physicians sometimes did not answer the questions due to time constraints or due to misled doctor-patient confidentiality concerns; or patients had already been discharged at the time of the case investigation often due to delayed notifications by the laboratory. Several LHAs suggested omitting clinical variables relating to symptoms or the focus of the infection as these were often laborious and time-consuming to collect.

When patients were hospitalized in another district than their place of residence, 32% (12 of 38) of the hospital-LHAs (LHA of the district where the hospital is located) conducted the case investigations in the hospitals themselves, 5% (2 of 38) decided on a case-by-case basis which LHA had to investigate, and 63% (24 of 38) forwarded the notification forms to the patient-LHA (LHA of the district of the patient’s residence) without their own investigations.

### Timeliness

#### Diagnosis to notification

All laboratories reported that they sent out notifications on the day the diagnosis was confirmed. However, one laboratory sent notifications forms by regular mail, which can take longer than 1 day. 86% (32 of 37) of LHAs reported that they received the notifications on the day of or the day following diagnosis. Analysis of surveillance data showed that median duration from diagnosis to the receipt of the notification at LHAs was 2 days (Table 2). Although delayed notifications were not specifically identified by LHAs as an issue in terms of implementation of control measures, some did report that this could mean that data collection in hospitals was difficult or impossible.

**Table 2:** Timeliness of notification and data transmission of notified MRSA cases; Germany 2009–2016

	Median [days]	IQR^a^ [days]
Diagnosis to notification (*n* = 1530)	2	1;4
Notification to data entry at LHA (*n* = 2245)	0	0;0
Data entry at LHA to data arrival at RKI (*n* = 2245)	1	1;2

^a^ IQR: interquartile range

#### Notification to data entry and data entry to arrival at RKI

Median duration from the receipt of a notification to its entry in the electronic system at the LHAs was less than 1 day and from data entry to arrival of data at RKI 1 day (Table 2). Data transmission from LHAs to SHAs within 24 h was reported by 60% of SHAs (6 of 10). Three SHAs reported that a few LHAs did not transmit data daily when there were staff shortages, when LHAs wanted the data to be complete before entering the case into the database, or when two LHAs were involved in investigations and forwarding of notification forms between LHAs was prolonged.

### Data quality

#### Data completeness

Data completeness of the 27,706 cases notified between 2009 and 2016 reached 96% to 100% for variables related to demographic characteristics and clinical presentation. Data completeness for diagnostic methods was 95% for the “sample type” for the whole period and improved markedly for “type of susceptibility testing” from 11% in 2009 to 95% in 2016. Apart from date of death, date variables were often not specified: the date of disease onset and the date of hospital admission had been specified for 68% and 80% of the cases respectively; and for only 56% both date variables had been specified. Completeness of additional information like symptoms and risk factors could not be assessed as a checkbox option “none” was not provided for these variables.

#### Data quality management

Quality checks were performed by 97% of LHAs (36 of 37) on the data entered into the database. All LHAs (36 of 36) tried to identify and delete duplicate notifications by either automatic or manual procedures. Additional quality checks were reported to be conducted by 72% of LHAs (26 of 36); most of them specified that the responsible SHAs checked for missing or implausible data. The majority of SHAs (70%; 7 of 10) reported that they performed these quality checks on every single case.

### Acceptability

**Fig. 3:**
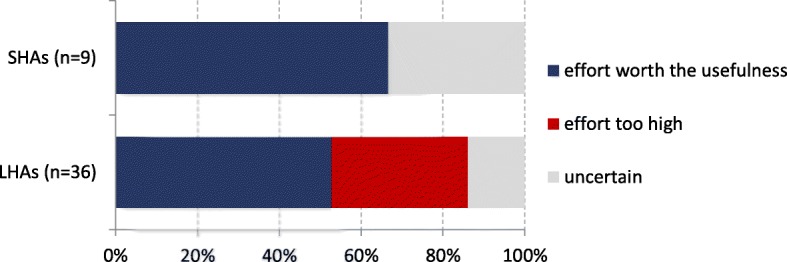
Balance of effort and usefulness of the surveillance system for invasive MRSA infections in Germany; interviews with SHAs and LHAs 2016/2017 (*n* = 45)

According to an analysis of the surveillance data, since 2009 all but one LHA had transmitted at least one case of invasive MRSA infection. Case investigations could not always be completed by 15% of LHAs (5 of 33). This was partially due to the fact that doctors felt that they would breach doctor-patient confidentiality if they provided the required information. Delayed notifications made it also more difficult to collect all the data as patient charts were no longer easily accessible. Some interviewees commented that the workload associated with the investigation of invasive MRSA infections was justified given the severity of the disease. Aggregated data was disseminated to interested parties by LHAs, but one laboratory stated that it would be useful to get feedback on the conclusions drawn from the data. Most LHAs (86%; 32 of 37) reported that they were able to collaborate well with hospitals. The majority of SHAs and LHAs stated that the benefits of the surveillance system were worth the workload associated with investigating cases of invasive MRSA infections (Fig. 3).

### Usefulness

According to LHAs and SHAs the usefulness of the system was partly due to the epidemiological data it delivered so that trends could be identified. Some respondents stated that notified invasive MRSA infections were a good proxy indicator for trends in all MRSA infections or even colonizations, conversely others voiced the opinion that the system only detected the “tip of the iceberg” and that the true burden could not be estimated using notified cases. Almost all interviewees (76% of LHAs and 90% of SHAs) analyzed their data, although some LHAs stated that they had too few cases to conduct any further analysis for MRSA only. Most interviewed sites (90%; 43 of 48) made use of published surveillance data, 48% using data from their federal state and 54% using national data published by RKI (multiple answers were possible). They either used the data for routine reports, for queries from press or policy makers, or to inform local networks for control of antimicrobial resistance.

**Fig. 4:**
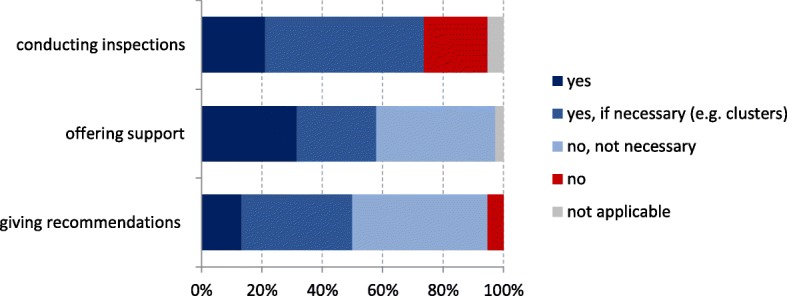
Actions taken by LHAs following notifications of invasive MRSA infections in Germany; interviews with LHAs 2016/2017 (*n* = 38)

Another benefit reported by LHAs was that LHAs took action following notifications and provided advice regarding control measures where required. However, some LHAs also reported that additional advice from their side was usually not required as they already knew which hospitals had hygiene guidelines and whether they were being followed. Some LHAs performed inspections of hospital wards when a case had been notified but most of them only did this in response to clusters of MRSA cases which rarely were invasive infections (Fig. 4). Two LHAs reported that they had detected hygiene deficiencies due to notified invasive MRSA infections. LHAs used case investigations following notifications to assess whether hospitals were complying with hygiene guidelines. This was reflected in the data collection forms, most of which (84%) contained questions regarding mode of transmission or prevention and control measures. Several LHAs reported that case investigations led to an intensified contact to hospitals, a better collaboration in terms of hygiene, and a better acceptance of prevention measures from the hospitals.

On a scale of 1 to 10 for the usefulness of the system, LHAs allocated a median of 7 points (range 2 to 10) and SHAs 6.75 points (range 2 to 10) to the surveillance system.

## Discussion

We conducted the first detailed evaluation of the German mandatory surveillance system for invasive MRSA infections including interviews with stakeholders and an analysis of surveillance data. We found that overall the aims of the system were met. However, by assessing the individual attributes of surveillance systems as recommended by the guidelines of the CDC [8], we also identified some areas for improvement (Table 3).

**Table 3:** Recommendations for improving statutory MRSA surveillance; Germany 2016/2017

Attribute assessed	Recommendations for improvement
Simplicity	• Reducing variables on clinical symptoms • Use data collection forms • Deposit data collection forms with IPC team of hospital • Clarify that hospitals are obliged legally to collaborate with LHAs on data collection
Data quality	• Clarify that hospitals are obliged legally to collaborate with LHAs on data collection • Include variables to be able to discriminate between hospital and community acquired infections
Timeliness	• None
Acceptability	• Reducing variables on clinical symptoms
Usefulness	• LHA with regulatory supervision should investigate and check on IPC measures in hospital • Include variables to be able to discriminate between hospital and community acquired infections

The MRSA surveillance aims to improve surveillance of healthcare associated infections and provides nationwide and mostly complete data on the epidemiology of invasive MRSA infections. This information is used to inform members of local networks for control of antimicrobial resistance, policy makers, and the public. The second aim, which is to support LHAs in preventing and controlling healthcare associated infections, is met by providing an additional opportunity for LHAs to check on measures undertaken in hospitals. MRSA surveillance had also led to a closer collaboration between LHAs and hospitals and might contribute towards better compliance with guidelines on infection prevention and control in hospitals.

However, when LHAs conduct case investigations in hospitals in other districts (because their resident is hospitalized in a different district), they cannot check on infection control measures in these hospitals as they have no regulatory supervision over these hospitals (according to the Infection Protection Act). In order to avoid this problem we recommend that LHAs of the district of the hospitals investigate and check on control measures if necessary and then forward the information to the patient-LHA for data transmission to the SHA. The results of this evaluation will inform the development of a new electronic reporting system with a particular focus on improving the exchange of data and the collaboration between LHAs.

Though the system was assessed to be useful, we found it to be complicated, resulting in a considerable workload for data collection by LHAs. Collecting data in hospitals was perceived to be labor intensive and time-consuming by many LHAs with almost half of the interviewed LHAs requiring more than 30 min for each case investigation. When the system was introduced, an average 26 min were estimated to be necessary for managing a case and 2000 cases were expected annually [4]. As currently about 3000 cases are notified annually, the resulting workload is considerably higher than estimated. Reducing the amount of data to be collected per case would address this. Several LHAs suggested that information on symptoms and focus of infection was unnecessary. They felt this information was difficult to ascertain and that it was not used by the LHAs. Omitting these variables might help making the system simpler and less resource-intensive and improve acceptability without reducing its usefulness.

Another approach to simplify data collection would be to provide the IPC team of the hospital with data collection forms which they could complete independently and submit to the LHAs. However, some LHAs considered speaking directly to the affected wards useful. LHAs will need to individually find a balance between the simplest way of data collection and the need for dialogue with the hospital.

A significant barrier to data collection was the refusal by some physicians to share detailed information due to concerns around patients’ data confidentially. In a response to this problem, the German law has recently been changed [13, 14]. Hospitals are now explicitly obliged to collaborate with LHAs on the collection of surveillance data. This will facilitate data collection for LHAs in the future.

Another issue that we identified was the inability to discriminate between hospital and community acquired MRSA infections for many of the notifications. This is due to low levels of data completeness for the dates of disease onset and of hospital admission, which are often used to identify hospital acquired infections. From the current surveillance system, it is also impossible to determine whether patients were transferred from other institutions where transmission might also have occurred, prior to hospitalization. We suggest this should be captured by the surveillance system, as it is for example the case in the English or US American system [15, 16].

This study had some limitations due to a possible selection bias. The selection of the LHAs was not random, but based on voluntary participation. However, 10% of LHAs were included in the study and their selection was purposefully diverse with the inclusion of small and large LHAs in rural and urban areas in Germany with low and high MRSA incidence. Due to voluntary participation, the results may not represent the view of less interested LHAs that might have more or less problems with the surveillance system than the interviewed sites. However, analysis of interviews with LHAs as well as SHAs showed that there were no more new aspects introduced during later rounds of interviews suggesting that data saturation had been reached. In addition, we presented the results to LHAs and SHAs at a conference and at meetings asking for their comments, which were generally consenting.

## Conclusions

In summary we found the surveillance system for invasive MRSA infections to be useful, timely, and well accepted with mostly good data quality and to be fulfilling its aims. Reducing the amount of information to be collected by LHAs could make the system simpler and less resource-intensive without impacting on its usefulness and may even increase its acceptance especially in LHAs.

## Abbreviations

CDC: Centers for Disease Control and Prevention
CSF: Cerebrospinal fluid
IPC: Infection prevention and control
IQR: Interquartile range
LHA: Local health authority
MRSA: Methicillin-resistant *Staphylococcus aureus*
RKI: Robert Koch Institute
SHA: State health authority
